# *In vitro* elution of amikacin, cefazolin, gentamicin, ampicillin/sulbactam, and meropenem from a commercially available calcium sulfate delivery kit

**DOI:** 10.3389/fvets.2024.1419769

**Published:** 2024-08-05

**Authors:** Elizabeth A. Maxwell, Taylor Howell, Rachel Mester, R. Avery Bennett, Crisanta Cruz-Espindola, Dawn Boothe

**Affiliations:** ^1^Department of Small Animal Clinical Sciences, University of Florida College of Veterinary Medicine, Gainesville, FL, United States; ^2^Department of Veterinary Clinical Sciences, Louisiana State University School of Veterinary Medicine, Baton Rouge, LA, United States; ^3^Department of Anatomy, Physiology and Pharmacology, Auburn University College of Veterinary Medicine, Auburn, AL, United States

**Keywords:** antibiotics, surgical site infections, calcium sulfate, plaster of Paris, beads, wound management, elution

## Abstract

**Introduction:**

The use of implantable antibiotic beads has become a frequent treatment modality for the management of surgical site infections in human and veterinary medicine. The objective of this study is to describe the elution kinetics of five antibiotics from a commercially available calcium sulfate antibiotic delivery kit. A secondary goal was to compare elution concentrations with minimal inhibitory concentrations (MIC) for commonly encountered bacteria from the University of Florida’s veterinary microbiology laboratory database.

**Methods:**

Calcium sulfate powder was combined with amikacin, cefazolin, gentamicin, ampicillin/sulbactam, and meropenem. Triplicates of three antibiotic-loaded beads were immersed in 5 mL of phosphate-buffered saline (PBS) and kept at 37°C under constant agitation. Antibiotic-conditioned PBS was sampled at 14 time points from 1-h to 30 days and analyzed by liquid chromatography to determine the antibiotic concentration.

**Results:**

All beads eluted concentrations of antibiotics for the 30-day sampling period, except for ampicillin/sulbactam, with the most antibiotics being eluted within the first week. The concentration of antibiotics within the eluent within the first 3–9 days (3- and 5-mm beads, respectively) was greater than the MIC of common isolates. The 5 mm bead samples were superior in maintaining higher concentrations for a longer period, compared to the 3-mm beads.

**Discussion:**

CSH beads eluted antibiotics over the 30-day course of the study. Most of the antibiotic elution occurred within the first week and was maintained above the MIC of commonly encountered isolates. This information may be useful for clinical decision making for treatment of local infections encountered in practice.

## 1 Introduction

The use of implantable antibiotic beads has become a frequent treatment modality for the management of both soft tissue and orthopedic infections in human and veterinary medicine (1–7). Placement of antibiotic beads into an infected site facilitates delivery of a high concentration of antibiotics locally, while reducing toxicity associated with long-term systemic administration of high doses of antibiotics (6, 8–10). This makes it possible to deliver and maintain an antibiotic concentration in local tissues above the minimal inhibitory concentration (MIC) of bacteria in the diseased tissue for extended periods by use of a single surgical application (2, 6, 11–13). Previous research has suggested that high concentrations of an antibiotic have the ability to infiltrate a biofilm (1, 14). For orthopedic periprosthetic joint infections (PJI), the formation of biofilm was determined to be the key virulence factor as it relates to their resistance to antibiotics (5, 15). McConoughey et al. and Fux et al. reported that the most common pathogens that formed biofilms in PJI were *Pseudomonas aeruginosa* and *Staphylococcus aureus* (5, 15, 16). A method that was researched and tested in effacing these biofilms was the application of antibiotic-loaded, synthetic calcium sulfate beads (CS-B). The CS-B, containing tobramycin and vancomycin, were placed into already flourishing biofilm colonies in controlled Petri and agar plates. With time, the CS-B was shown to prevent bacterial colonization and reduce lawn biofilms grown on the plates drastically (5, 17, 18).

Calcium sulfate has been considered an ideal delivery vehicle for antibiotics, as it is biodegradable, cost effective, and aids in filling dead space (19). It also decreases the potential risk of inflammatory reactions that can result from implantation of inert foreign material and does not require removal via a secondary surgical procedure, as do many other antibiotic delivery vehicles, such as polymethylmethacrylate (PMMA) (2, 6, 11–13). It has been known to be osteoconductive when placed in areas of osteomyelitis and fracture sites, providing a basis for new bone formation (2, 7, 11–13, 20). As the calcium sulfate beads dissolves, there is sustained release of antibiotic over time.

The primary objective of this study was to (1) describe the *in vitro* elution kinetics of five antibiotics (amikacin, cefazolin, gentamicin, ampicillin/sulbactam, and meropenem) from a commercially available calcium sulfate local antibiotic delivery kit over 30 days and (2) to compare elution concentrations with Clinical Laboratory Standards Institute’s (CLSI) MIC for commonly encountered bacteria from the University of Florida’s veterinary microbiology laboratory database. The hypothesis is that a biphasic elution of antibiotic is expected in all antibiotics with maximum release in the initial 24 h followed by gradual release that occurs concurrently with dissolution of the calcium sulfate beads over 30 days. Additionally, the authors hypothesize that concentrations of antibiotics in the eluent will be greater than the MICs for commonly encountered bacteria.

## 2 Materials and methods

### 2.1 Bead preparation

All antibiotic-impregnated and control beads were created using a novel kit of resorbable high purity alpha hemihydrate calcium sulfate (Kerrier Local Antibiotic Delivery, Palm Beach Garden, FL, United States). This kit is marketed specifically for veterinary medicine and use in local delivery of antimicrobials. Pharmaceutical-grade calcium sulfate alpha-hemihydrate powder (CSH) and antibiotics were mixed according to the manufacturer’s guidelines, forming a paste. 15 g of CSH was mixed with the following amount of antibiotics separately: 500 mg of amikacin (West-Ward Pharmaceutical, now Hikma Pharmaceuticals USA Inc., Berkeley Heights, NJ) with 2 mL of mixing solution (saline), 1 g of cefazolin powder (Apotex Inc., Weston, FL) with 4 mL of mixing solution, 400 mg of gentamicin liquid (VetOne, Boise, ID), 750 mg of ampicillin/sulbactam powder (Mylan Institutional LLC, Rockford, IL) with 4 mL of mixing solution, and 500 mg of meropenem powder (Auromedics Pharma LLC, East Windsor, NJ) with 4 mL of the mixing solution. A control group was also prepared using 15 g of CSH and 4 mL of mixing solution. Standard and nonstandard instructions, provided by Kerrier LLC, were followed according to the type and formulation of the antibiotic. Each antibiotic mixture was mixed thoroughly for 30–60 s until a paste was formed. The paste was spread into the 3- and 5-mm cavities in each mold from the kit. The paste was spread smoothly over the mold to ensure an adequate amount of paste was used to produce a bead shape (hemisphere). Controlled tapping of the molds on a table surface was implemented to reduce air trapping in the cement. Molds were set aside to allow beads to harden (between 20 and 50 min) according to the manufacturer’s instructions.

### 2.2 Elution testing

After the beads were set, they were procured by bending the mold and allowing the beads to fall out. The beads were separated based on their size (3- and 5-mm). Eighteen beads were chosen from each batch and weighed. Beads that were not fully formed or had deficits from air pockets were excluded. Similar weight beads were placed in six sterile 10 mL tubes in triplicates (three from the 3-mm mold, three from the 5-mm mold) for each antibiotic and the control group. The antibiotic-loaded and control beads were immersed in 5 mL of phosphate-buffered saline (PBS) and maintained at 37°C under constant agitation with an orbital shaker at 100 rpm. Antibiotic-conditioned PBS was sampled at 14 time points (1, 3, 6, 12, 24 h; 3, 6, 9, 12, 15, 18, 22, 26, 30 days). At each time point, 5 mL of PBS were extracted from each tube and placed into 5 mL cryovials for further analysis. The cryovials were placed in the freezer at −80°C until they were analyzed at the end of the study. The tubes were replenished with the 5 mL of sterile PBS and placed back under constant agitation in the orbital shaker. The pipettes were changed with each antibiotic and between each bead size within each antibiotic, except for the control group. High-performance liquid chromatography (HPLC) was used to measure the antibiotic concentration in the eluent samples, apart from amikacin and gentamicin. Samples were prepared in triplicate and concentrations were averaged.

### 2.3 Antibiotic analysis

Beta lactam-antibiotics (cefazolin, ampicillin, sulbactam, and meropenem) were analyzed by HPLC with ultraviolet (UV) detection. The method was a modification of a protocol previously described (21–23). The HPLC system consisted of an Alliance Waters 2695 separation module (Waters Corporation™, Milford, MA, United States), and a Waters 2489 Dual λ Absorbance Detector (Waters Corporation™, Milford, MA, United States). Separation was achieved with a XSelect CSH C18, 5 μm, 4.6 mm × 150 mm chromatographic column (Waters Corporation™, Milford, MA, United States) (21–23), protected by a Sunfire C18, 5 μm, 4.6 mm × 20 mm guard cartridge (Waters Corporation™, Milford, MA, United States). The mobile phase consisted of 50 mM sodium dihydrogen phosphate dihydrate pH 2.4 Buffer: Acetonitrile (VWR®, Radnor, PA, United States) (21) 90:10 v/v, 85:15 v/v, and 80:20 v/v for ampicillin sodium/sulbactam sodium, meropenem, and cefazolin, respectively, with a flow rate set to 1.5 mL/min. The standard curves for the beta lactam-antibiotics were generated ranging from 25 to 500 μg/mL, and from 1 to 15 μg/mL by fortifying PBS 1X with known amounts of meropenem (USP™, Rockville, Maryland, United States), ampicillin, sulbactam, and cefazolin (Sigma-Aldrich® company, Round Rock, TX, United States) reference standards. The standard curves were accepted if the coefficient of determination (*r*^2^) was at least 0.999 and the predicted concentrations were within ±10% of the actual concentrations. The sample was monitored at 210 nm (21–23). Once the beta lactam-antibiotic samples were received, they were thawed and 1.5 mL of solution was centrifuged at 16,000 × *g* for 5 min at room temperature, and then 25 μL of the supernatant was injected into the HPLC System in duplicate.

The HPLC assay was validated for beta lactam-antibiotics in beads. The linear correlation coefficient for all of them was at least 0.999, the limit of detection (LOD) for the beta lactam-antibiotics was 0.5 μg/mL. The lower limit of quantification (LOQ) for the beta lactam-antibiotics was 1 μg/mL. The precision (CV %) for cefazolin, ampicillin, sulbactam, and meropenem in PBS 1X was measured in the concentration range of 1 to 500 μg/mL, and the results were between 0.1 and 14.9%. The accuracy (CV%) for cefazolin, ampicillin, sulbactam, and meropenem in PBS 1X was measured in the concentration range of 1–500 μg/mL, and the results were between 0.2 and 0.08%. The % recovery in PBS 1X was measured in the concentration range of 1–500 μg/mL, and the results were between 92 and 103.2%.

Amikacin and gentamicin were detected and quantitated in PBS 1X using a Siemens Dimension® Xpand® Plus Integrated Chemistry System (Siemens, Malvern, PA, United States). Amikacin was detected and quantitated using a Siemens Syva® Emit® homogenous enzyme immunoassay (10445383). The assay is calibrated in reconstituted lyophilized human serum with concentrations ranging from 2.5 to 50 μg/mL. Gentamicin was detected and quantitated using a Siemens Dimension® GENT gentamicin particle enhanced turbidimetric inhibition immunoassay (PETINIA) (10444927). The assay is calibrated in a synthetic bovine serum albumin matrix using the Siemens Dimension® DRUG Calibrator II (10445005) with concentrations ranging from 0.0 to 12.7 μg/mL. Prior to analysis of samples, both assays were tested using Thermo MAS™ PAR™ TDM controls (PTD1-1001) and confirmed to be in working order. Gentamicin and amikacin samples exceeding the assay’s upper limit of quantitation (12.7 and 50 μg/mL, respectively) were repeated at a dilution to bring the concentration within the assay range.

For the gentamicin, the precision (CV%) was 2.74%, and the linear correlation coefficient was 0.997. For the amikacin, the precision (CV%) at the 10 μg/mL and the 15 μg/mL was 8.6%, and 3.9, respectively. The correlation coefficient was 0.961.

### 2.4 Mean inhibitory concentrations

Clinical Laboratory Standards Institute MICs for commonly encountered bacteria, such as *Staphylococcus pseudintermedius* (MRSP and MSSP), *Escherichia coli*, and *Pseudomonas aeruginosa*, were obtained from the University of Florida College of Veterinary Medicine’s microbiology laboratory database. The MIC of these organisms was compared to the concentrations of the following antibiotics used in our study: amikacin, cefazolin, and gentamicin, to determine their MIC breakpoints. The MICs for meropenem and ampicillin/sulbactam have not been established.

### 2.5 Statistical analysis

All elution testing was performed in triplicate. Data that followed a normal distribution was presented as mean (standard deviation), and non-normal data was presented as median (range).

## 3 Results

**Figure 1:**
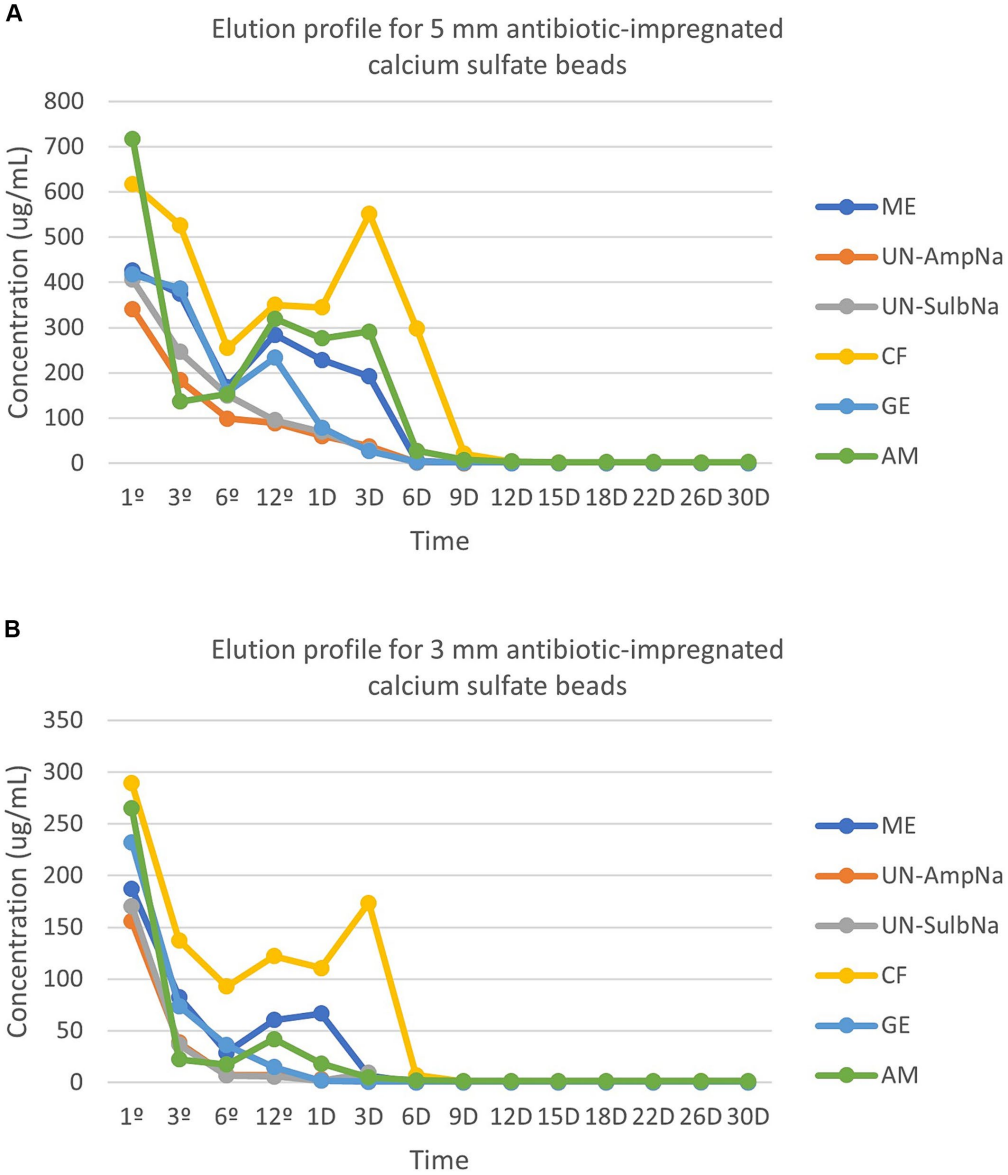
**(A)** The elution profile for five antibiotics [amikacin (AM), cefazolin (CF), gentamicin (GE), ampicillin (UN-AmpNA)/sulbactam (UN-SulbNA), and meropenem (ME)] impregnated in 5-mm calcium sulfate beads. The elution concentration was measured over a 30-day period. All antibiotic impregnated CSH beads eluted detectable concentrations of antibiotics for the 30-day sampling period except for ampicillin/sulbactam, which eluted detectable concentrations of antibiotics for 9 days. **(B)** The elution profile for five antibiotics [amikacin (AM), cefazolin (*CF*), gentamicin (GE), ampicillin (UN-AmpNA)/sulbactam (UN-SulbNA), and meropenem (ME)] impregnated in 3-mm calcium sulfate beads. The elution concentration was measured over a 30-day period. All antibiotic impregnated CSH beads eluted detectable concentrations of antibiotics for the 30-day sampling period except for ampicillin/sulbactam, which eluted detectable concentrations of antibiotics for 3 days.

Peak concentrations (C_max_) occurred at 1-h for both sizes with all antibiotics. The following are the peak concentrations in μg/mL for each antibiotic for the 3- and 5-mm beads, respectively: amikacin (264.80, 717.20), cefazolin (289.38, 617.60), gentamicin (232.17, 417.30), ampicillin (155.69, 341.48), sulbactam (170.39, 390.81), and meropenem (187.27, 462.18) (Figures 1A,B).

Elution of amikacin, gentamicin, and the 5-mm beads of meropenem followed a biphasic pattern. A second peak in concentration for amikacin occurred at hour 12 for both sizes (3-mm beads 41.92 μg/mL, 5-mm beads 320.10 μg/mL). Gentamicin’s second peak in concentration occurred at hour 12 for the 5-mm beads (233.53 μg/mL), but a second peak was not observed in the 3-mm beads. Meropenem had a second peak in concentration at hour 12 for the 5 mm-bead (284.51 μg/mL). There was a triphasic pattern for the 3-mm beads of meropenem and both sizes of beads for cefazolin. The 3-mm beads of meropenem peaked a second time at hour 12 (60.39 μg/mL), then again on day 1 (66.54 μg/mL). The second peak for cefazolin occurred at hour 12 for both sizes (3-mm beads 122.25 μg/mL, 5-mm beads 350.59 μg/mL). The third peak of cefazolin occurred on day 3 for both sizes (3-mm beads 173.67 μg/mL, 5-mm beads 551.40 μg/mL). Ampicillin and sulbactam both displayed a monophasic pattern with the C_max_ occurring at hour 1 as described above. No drug elution occurred from the control beads.

Minimal inhibitory concentrations breakpoints for amikacin, cefazolin, and gentamicin were compared to the susceptibility breakpoints of *Staphylococcus pseudintermedius* (MRSP and MSSP), *Escherichia coli*, and *Pseudomonas aeruginosa*. The concentration of antibiotics within the eluent within the first 3–9 days (3 and 5 mm beads, respectively) was greater than the MIC of common isolates encountered by the University of Florida’s Veterinary Diagnostic Lab (Table 1).

**Table 1:** Minimum inhibitory concentration (MIC; μg/mL) of isolates commonly encountered by the University of Florida College of Veterinary Medicine Diagnostic Laboratory.

Drug name	*S. pseudintermedius* (MSRP)	*S. pseudintermedius* (MSSP)	*E. coli*	*P. aeruginosa*
	*N* = 914	*N* = 722	*N* = 1,596	*N* = 582
Amikacin	16.00	16.00	4.00	4.00
Cefazolin	2.00	2.00	2.00	64.00
Gentamicin	4.00	4.00	0.50	<2.00

Data not available for meropenem and ampicillin/sulbactam (Unasyn). MIC presented is the most frequently represented distribution.

## 4 Discussion

This study characterizes the elution kinetics of five antibiotics from a commercially available calcium sulfate local antibiotic delivery kit. While most antibiotics were eluted within the first week, antibiotics were detectable in small amounts in all samples up to 30 days, except for ampicillin/sulbactam. Most of the antibiotics followed a biphasic or triphasic pattern. Five-millimeter beads samples were superior in maintaining higher concentrations for a longer period of time compared to the 3-mm beads for all analyzed antibiotics.

The differences in the elution profiles between the sizes of beads are unclear but may be attributed to several factors. Aiken, et al. suggests that different sizes of beads have different surface-to-volume ratios. While this would mean that smaller beads have a larger surface area, contributing to the reduced diffusion path from the center to the surface for each antibiotic bead, in this study, larger beads appeared to load more antibiotics and therefore maintain higher concentrations. But the solubility of the antibiotic, molecular weight, and potential for binding to calcium sulfate must also be considered (1).

Changes in concentration over time of locally administered antibiotics vary according to the type and stability of the drug and its substrate; the likelihood of an exothermic reaction to occur when mixing drug and substrate, the size, shape, porosity, and degradation habits of the bead; the rate and volume of fluid flowing around the bead; the vascularity of the tissues in which the beads have been implanted; and the diffusion characteristics of the antibiotics (6, 8, 24–27). While antibiotic concentrations remained above the MIC during the first week for most of the antibiotics in this study, only the elution kinetics of three beads per size were evaluated. Clinically, up to 10 times that number of beads are commonly used for the treatment of infections. While this may be excessive in terms of the MIC needed to accomplish bacterial death, elution times above the MIC may be much higher with a longer duration than reported in this *in vitro* study.

Previously, researchers have concluded that time-dependent drugs, such as cefazolin, are more efficacious when concentrations are stable between 2 and 5 times above the MIC for at least 50% of the dosing intervals because it improves antimicrobial efficacy against certain pathogens with an MIC close to the breakpoint (6, 28–31). For concentration-dependent antibiotics, like amikacin and gentamicin, it has been shown that concentrations between 10 and 12 times the MIC can improve antimicrobial penetration of biofilm and increase the killing of the bacteria (6, 28, 32, 33). Susceptibility breakpoints may be helpful in predicting the effectiveness or a particular antibiotic administered systemically, however it remains unknown how this information can be applied to locally administered antibiotics.

The experimental parameters used in this study were performed to mimic the intra-and postoperative environments in a relatively stable atmosphere. The sampling period of 30 days was chosen for the study as that is the reported time frame by which the beads would be degraded and is consistent with other studies (6, 7, 34–37). Furthermore, it is important to note that while the beads will begin to dissolve once they are placed into solution, thereby releasing antibiotics into the buffer, agitating the samples and exchanging the buffer solution at specific time points, promotes elution of the antibiotics and thereby mimicking an *in vivo* environment.

There are several limitations to this study. The *in vitro* setting does not account for the complexity of a tissue infection, interactions between bacteria, the host immune responses, and the formation of a biofilm. *In vitro* models do not consider multiple factors such as blood clot formation, which will affect the diffusion of the antibiotic into the surgical site, and protein binding, which will affect the release of the antibiotics into the extracellular matrix which affects efficacy (1, 38, 39). Factors such as absorption, distribution, metabolism, and excretion cannot be accurately assessed in an *in vitro* setting. These factors play a crucial role in determining the drug’s efficacy and safety profile in humans. Therefore, the results from this *in vitro* study may not fully reflect the real-world response to infection.

Additionally, the antibiotics selected for this study were selected due to their broad-spectrum activity against a variety of bacteria commonly encountered in veterinary practice. However, it is important to acknowledge that the World Health Organization’s classifies meropenem as a critically important antimicrobial in human medicine. While all antibiotics, except for ampicillin/sulbactam, were eluted at concentrations above the MIC for common isolates for at least 3–9 days, there was a decline in concentrations over time, potentially leading to subtherapeutic levels toward the end of the 30-day period. This raises concerns about the potential development of resistance, particularly when using carbapenems like meropenem. To mitigate these risks, future research could focus on strategies for selecting antibiotics that minimize the risk of resistance development, or methods for maintaining therapeutic concentrations of antibiotics over longer periods, such as is the case when using a higher number of beads. These considerations are crucial for the responsible use of antibiotics in veterinary practice and for the broader goal of antimicrobial stewardship.

In conclusion, CSH beads impregnated with amikacin, cefazolin, gentamicin, and meropenem eluted antibiotics over the 30-day course of the study with most of the antibiotic elution occurring within the first week. Larger beads maintained higher concentrations for a longer period, consistent with a previous study (40). Ampicillin/sulbactam was not detectable beyond 9 days in this study using three beads; however, does not likely represent the tissue fluid concentrations achievable when using a higher number of beads. The information from our study may be useful for clinical decision making when implementing antibiotic beads for treatment of local infections and *in vivo* studies are warranted.

## Author’s note

Presented in part as an abstract at the American College of Veterinary Surgeons Surgery Summit, Portland, October 2022.

## Data availability statement

The original contributions presented in the study are included in the article/supplementary material, further inquiries can be directed to the corresponding author.

## Author contributions

EM: Data curation, Investigation, Project administration, Resources, Supervision, Writing – original draft, Writing – review & editing. TH: Data curation, Formal analysis, Investigation, Methodology, Writing – original draft, Writing – review & editing. RM: Data curation, Investigation, Writing – original draft, Writing – review & editing. RB: Conceptualization, Supervision, Writing – original draft, Writing – review & editing. CC-E: Conceptualization, Data curation, Formal analysis, Investigation, Methodology, Writing – original draft, Writing – review & editing. DB: Conceptualization, Data curation, Formal analysis, Methodology, Writing – original draft, Writing – review & editing.
